# Case report: A novel patient presenting TRIM32-related limb-girdle muscular dystrophy

**DOI:** 10.3389/fneur.2023.1281953

**Published:** 2024-01-18

**Authors:** Martina Rimoldi, Gloria Romagnoli, Francesca Magri, Sara Antognozzi, Claudia Cinnante, Elena Saccani, Patrizia Ciscato, Simona Zanotti, Daniele Velardo, Stefania Corti, Giacomo Pietro Comi, Dario Ronchi

**Affiliations:** ^1^Neuromuscular and Rare Disease Unit, Fondazione IRCCS Ca' Granda Ospedale Maggiore Policlinico, Milan, Italy; ^2^Neurology Unit, Fondazione IRCCS Ca' Granda Ospedale Maggiore Policlinico, Milan, Italy; ^3^Department of Radiology, Istituto Auxologico Italiano, IRCCS, Milan, Italy; ^4^Neurology Unit, Department of Specialized Medicine, University Hospital of Parma, Parma, Italy; ^5^Dino Ferrari Center, Department of Pathophysiology and Transplantation, University of Milan, Milan, Italy

**Keywords:** LGMDR8, TRIM32, limb-girdle muscular dystrophy, clinical exome sequencing, tripartite motif-containing proteins

## Abstract

Limb-girdle muscular dystrophy autosomal recessive 8 (LGMDR8) is a rare clinical manifestation caused by the presence of biallelic variants in the *TRIM32* gene. We present the clinical, molecular, histopathological, and muscle magnetic resonance findings of a novel 63-years-old LGMDR8 patient of Italian origins, who went undiagnosed for 24 years. Clinical exome sequencing identified two *TRIM32* missense variants, c.1181G > A p.(Arg394His) and c.1781G > A p.(Ser594Asp), located in the NHL1 and NHL4 structural domains, respectively, of the TRIM32 protein. We conducted a literature review of the clinical and instrumental data associated to the so far known 26 *TRIM32* variants, carried biallelically by 53 LGMDR8 patients reported to date in 20 papers. Our proband's variants were previously identified only in three independent LGMDR8 patients in homozygosis, therefore our case is the first in literature to be described as compound heterozygous for such variants. Our report also provides additional data in support of their pathogenicity, since p.(Arg394His) is currently classified as a variant of uncertain significance, while p.(Ser594Asp) as likely pathogenic. Taken together, these findings might be useful to improve both the genetic counseling and the diagnostic accuracy of this rare neuromuscular condition.

## 1 Introduction

The term limb-girdle muscular dystrophy (LGMD) refers to typically non-syndromic childhood- and adult-onset group of muscular dystrophies, affecting primarily skeletal muscles, and usually associated with elevated serum creatine kinase (CK) concentration (1, 2). Patients with LGMD suffer from progressive muscle weakness and wasting, involving proximal more than distal districts, in particular muscles of the shoulder and pelvic girdles (1, 2). However, other muscle groups, such as facial, distal upper and lower limbs, may also be affected (3). In the pre-molecular era, LGMD diagnosis used to be purely clinical, and it could only be confirmed differentially once specific protein testing became available (4, 5) to exclude X-linked recessive neuromuscular disorders, such as Duchenne muscular dystrophy and Becker muscular dystrophy (1). Since the advent of molecular myology, pathogenic variants in 29 genes have been reported in distinct LGMD clinical presentations, classified into dominant (LGMDD) or recessive (LGMDR) forms according to the pattern of inheritance, with some genes presenting both kinds of transmission (1, 2).

LGMDR8 (MIM #254110) represents the subtype 8 of the autosomal recessive type of LGMD, previously known as LGMD2H (1, 2), and initially referred to as Sarcotubular Myopathy when first described in the Hutterite population of Manitoba, in North America (6–9). This subtype of LGMDR can display clinical heterogeneity, with symptom onset ranging between the first and the fourth decade of life (10). The clinical course is progressive, and severity goes from absence of symptoms to muscular weakness with atrophy (11, 12), possibly requiring the use of a wheelchair.

Biallelic variants in the *TRIM32* gene, on chromosome 9q33, have been associated with LGMDR8, which is caused by the homonymous protein deficiency (2). *TRIM32* encodes the member 32 of the TRIpartite Motif-containing (TRIM) ubiquitin E3 ligase family, which ubiquitinates several muscle substrates, including sarcomeric proteins (13). However, the specific effect on the muscle of the so far identified *TRIM32* variants has not been fully clarified yet. Bioinformatic modeling suggests these variants may cause a potential misfolding of the TRIM32 protein, especially at the level of the C-terminal domain, leading to pathogenic consequences on the muscle physiology (14). Beside the first variant – c.1459G > A, p.(Asp487Asn) – identified in the Hutterite population with a founder effect (6, 7, 9), additional 25 variants of *TRIM32* have been described to date in other 41 non-Hutterite LGMDR8 patients, worldwide (11, 13, 15–28).

**Figure 1:**
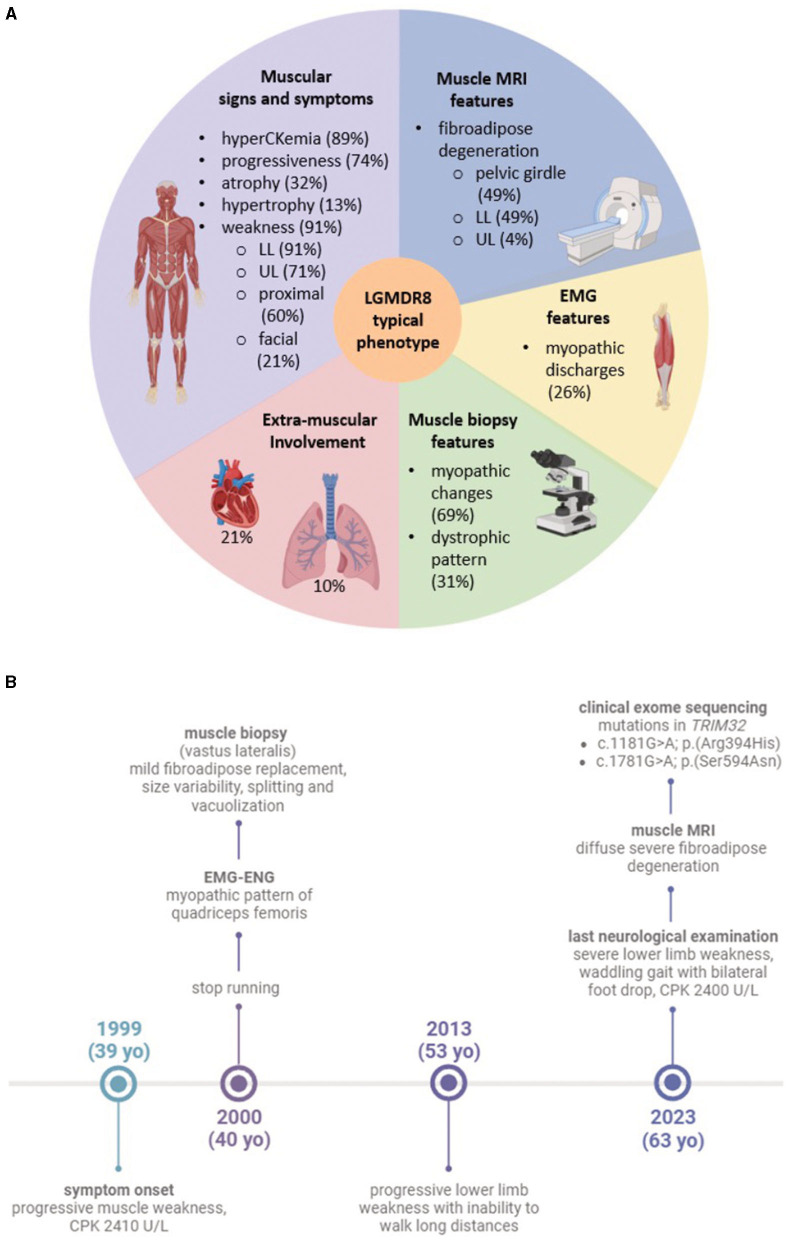
**(A)** Graphic of the typical LGMDR8 phenotype. The five sections summarize the most frequent clinical and instrumental features, drawn from the analysis of the 53 LGMDR8 *TRIM32*-mutated patients (including our case) reported in the literature; **(B)** timeline highlighting our patient's clinical history and diagnostic work-up. Figures **(A, B)** were created with BioRender.com.

Here, we present the novel case of an Italian 63-years-old man, who experienced progressive muscle weakness since the age of 39, and only received a molecular diagnosis 24 years later. He is the first LGMDR8 case in literature to be identified as compound heterozygous for two *TRIM32* missense variants, whose pathogenicity has not been clarified yet. We describe his clinical, histopathological, muscle magnetic resonance imaging (MMRI) and molecular findings, against the backdrop of other 52 LGMDR8 patients harboring biallelic *TRIM32* variants (Table 1; Figure 1A), reported across 19 published studies (7, 11, 13, 15–30).

**Table 1:** Summary of the 26 TRIM32 variants carried biallelically by the 53 LGMDR8 patients (including our case) reported in the literature.

**Variant**	**ACMG Class**	**Protein domain**	***N***	**Age at onset (years)**	**Age at last visit (years)**	**CPK (U/L)**	**Neuromuscular semiology**	**MMRI**	**EMG/ ENG**	**Muscle biopsy**	**Cardio-pulmonary involvement**	**Notes on ethnicity/family**	**Reference**
c.35dupA p.(Asp12Glufs^*^44)	n/a	pre-RING	1	25	49	1,684	LL > UL and P weakness; progressive; cleft lip and palate; bilateral pes cavus; lumbar lordosis	FR	M and NG	M	Normal	Indian; with BR parents	Chandrasekharan et al. (19)
c.59G > T p.(Cys20Phe)	3	RING	1	25	30	1,708	LL > UL and G weakness; C pseudohypertrophy	n/p	n/p	n/p	n/a	n/a	Chakravorty et al. (17)
c.115_116insT p.(Cys39Leufs^*^17)	n/a	RING	3	30–40	30–40	n/a	dLL and P weakness (3); BBs signs (2)	FR	n/p	D (1); n/p (2)	Normal	Spanish/Australian family; with BR parents	Servián-Morilla et al. (26)
c.459_462dup p.(Arg155Asnfs^*^29)	5	CC	1	10	40	1,450	LL, pUL, F and G weakness; progressive; WB since 30	n/a	n/p	M	Normal	Turkish	Johnson et al. (21)
c.467T > C p.(Leu156Pro)	3	CC	2	27; 36	43; 54	926; 1,500	LL, pUL and G weakness (2); progressive (2); muscle atrophy and WB at 53 (1); pseudohypertrophy (1)	n/p	n/p	M	n/a	Brothers	Mair et al. (22)
c.488T > C p.(Leu163Pro)	3	CC	2	3; 32	21; 40	6,500; 398	L and G weakness (2); progressive (2); WA at 20 (1)	atrophy and FR	n/p	M; D	Normal (1); n/a (1)	Pakistani (1); Persian (1)	Johnson et al. (21)
c.574dup p.(Glu192Glyfs^*^7)	5	CC	2	30; 30	56; 48	802; 443	L, G, A and PS weakness; WB since late 40s	atrophy and FR	n/p	D	Mild aberrant contraction (1)	Belgian	Johnson et al. (21)
c.650A > G p.(Asn217Ser)	3	inter CC-NHL1	4	20–67	57–67	n/a	L and G weakness (3); asymptomatic (1); FD (1); progressive (4); WA at 58 (1)	focal FR	n/p	D (2); n/p (2)	Normal	Spanish/Australian family	Servián-Morilla et al. (26)
c.691del p.(Ala231Glnfs^*^21)	5	inter CC-NHL1	1	early 30s	42	1,844	P and LL weakness; progressive	atrophy and FR	n/p	D	Normal	British	Johnson et al. (21)
c.872T > G p.(Ile291Ser)	3	inter CC-NHL1	1	19	48	120	L and G weakness; progressive; WA at 42	atrophy and FR	n/p	M	Normal	Persian	Johnson et al. (21)
c.1108del p.(Met370Cysfs^*^10)	4	NHL1	1	early 30s	42	1,844	P and LL weakness; progressive	atrophy and FR	n/p	D	Normal	British	Johnson et al. (21)
c.1163C > T p.(Ala388Val)	3	NHL1	2	30; 30	56; 48	802; 443	L, G, A and PS weakness; WB since late 40s	atrophy and FR	n/p	D	Mild aberrant contraction (1)	Belgian	Johnson et al. (21)
c.1181G > A p.(Arg394His)	4	NHL1	3	45; 30s; 39	53; 64; 63	500; n/a; 600; 2,400	pL (3), G (3), and NF (1) weakness; pL (2) and LL (1) atrophy; retractions (1); myalgia (2); paresthesia (1); WS (2); FD (1); progressive (3); WB since 60s (1); WA at 50 (1) and 63 (1)	n/a (1); n/p (1); atrophy and FR (1)	n/p (2); M (1)	D (1); M (2)	RBBB (1); LV hypertrophy (1); FVC 41% (1)	British (1); Italian (2)	Johnson et al. (21) (1); Saccone et al. (11) (1); our case
c.1184T > C p.(Ile395Thr)	3	NHL1	1	14	65	125	pL and G weakness; progressive	n/p	n/p	n/p	Aortic valve stenosis; RI and NIV	with BR parents	Ten Dam et al. (27)
c.1459G > A p.(Asp487Asn)	5	NHL3	14	0-8 (5); 20-31 (5); n/a (4)	7–15 (7); 23; 33–40 (5); 54	117–276 (5); 1,055–2,030 (5); 5–20x (3); n/a (1)	L (10), G (8), NF (4), F (2) and A (2) weakness; asymptomatic (4); pL atrophy (4); C pseudohypertrophy (2); C atrophy (1); +Gower's sign (2); myalgia and fatigue (3); WS (1); progressive (4); WB at 53 (1)	n/p (12); atrophy and FR (2)	n/p (13); M (1)	M (7); D (1); n/p (6)	n/a (9); normal (5)	German brothers (2); Hutterite brothers (2); Hutterite family (7); Bosnian Serbian (1); Serbian (1); Hutterite (1)	Schoser et al. (29) (4); Frosk et al. (7) (7); Johnson et al. (21) (2); Liewluck et al. (30) (1)
c.1560delC p.(Cys521Valfs^*^13)	5	inter NHL3-NHL4	3	< 10 (2); 37	40; 36; 44	860; 460; 1–2.5x	myalgia and paresthesia (2); L, G and F weakness (3); WB at 40 (2); F and pL atrophy (3); C pseudohypertrophy (2)	n/p (3)	M and NG	M (2); n/p (1)	Respiratory involvement (1)	Sweden (2); Croatian (1)	Borg et al. (15) (2); Saccone et al. (11) (1)
c.1603delC p.(Leu535Serfs^*^21)	5	inter NHL3-NHL4	1	30	40	400	LL > UL and P weakness; WS; WA at 40	n/p	M	M	n/a	non-Hutterite	Nectoux et al. (23)
c.1700A > G p.(His567Arg)	3	NHL4	2	24; 27	30	610; 427	L, G and NF weakness; progressive; fatigue; LL hypotrophy; +Gowers' sign	atrophy and FR	M	M	n/a	Chinese sisters	Guan et al. (20)
c.1701_1703del p.(Phe568del)	3	NHL4	4	20–67	57–67	n/a	L and G weakness (3); asymptomatic (1); FD (1); progressive (4); WA at 58 (1)	focal FR	n/p	D (2); n/p (2)	Normal	Spanish/Australian family	Servián-Morilla et al. (26)
c.1753_1766dup p.(Ile590Leufs^*^38)	5	NHL4	1	25	52	744	L and G weakness; myalgia; progressive	n/p	M	M	Normal	Turkish; with BR parents	Cossée et al. (16)
c.1771G > A p.(Val591Met)	2	NHL4	3	teens	n/a	n/a	FD first sign (3); L and G weakness (3); ankle contractions (3); progressive (3); WA at 30 (1)	diffuse FR	n/p	D (2); n/p (1)	Normal	Spanish/Australian family (3); with BR parents (1)	Servián-Morilla et al. (26)
c.1781G > A p.(Ser594Asn)	3	NHL4	2	46; 39	66; 63	4x; 2,400	L and G weakness (2); scapular G (1), pL and LL (1) atrophy; C pseudohypertrophy (1); rigid spine (1); WS (1); FD (1); myalgia (1); progressive (2); WA at 63 (1)	diffuse atrophy and FR	n/p (1); M (1)	M	Normal	Italian	Panicucci et al. (25); our case
c.1786C > G p.(Arg596Gly)	3	NHL4	1	14	42	≤ 2,000	L, PS and P weakness; progressive; scoliosis; joint laxity; WA since mid-30s	n/a	n/p	M	Atrial septal aneurysm	Italian	Johnson et al. (21)
c.1837C > T p.(Arg613^*^)	4	NHL5	2	34; 25	45; 35	317; 2x	pL, G and A weakness; progressive	n/a (1); atrophy and FR (1)	n/p (1); M (1)	M (2)	Mitral and tricuspid valve regurgitation (1)	Polish (1); n/a (1)	Johnson et al. (21) (1); Neri et al. (24) (1)
c.1855C > T p.(Pro619Ser)	3	NHL5	1	school age	28	746.81	pL, G and T weakness; pL atrophy; progressive; +Hoover's sign	atrophy and FR	M	n/p	LV contraction alteration	Azerbaijani	Marchuk et al. (13)

ACMG class, TRIM32 structural domain, clinical phenotypes, and instrumental findings, are provided for each listed variant. Please note that compound heterozygous patients appear twice (hence N > 53). The color coding refers to the TRIM32 structural domains and intermediate regions displayed in Figure 3B. A, abdominal; ACMG, American College of Medical Genetics and Genomics; BBs, Bardet-Biedl syndrome; BR, blood-related; C, calve; CC, coiled-coil; CPK, creatine phosphokinase; d, distal; D, dystrophic; EF, ejection fraction; EMG/ENG, electromyography/electroneurography; F, facial; FD, foot drop; FR, fatty replacement; FVC, Forced Vital Capacity; G, girdle; L, limb; LV, left ventricle; M, myopathic; MMRI, muscle magnetic resonance imaging; N, number of patients; NF, neck flexor; NG, neurogenic; NIV, non-invasive ventilation; n/a, not available; n/p, not performed; p, proximal; P, pelvic; PS, paraspinal; RBBB, right bundle branch block; RI, respiratory insufficiency; T, tongue; UL/LL, upper/lower limb; WA, walking aids; WB, wheelchair bound; WS, winged scapula.

## 2 Methods

The study was approved by the institutional review board of the Fondazione IRCCS Ca' Granda Ospedale Maggiore Policlinico, Milan (Italy). The patient provided written informed consent for all aspects of the study. All the details related to data collection and analysis are available in Supplementary material. Clinical, histological, immunohistochemical, MMRI, and molecular data from the proband were integrated within our literature review of additional 52 LGMDR8 patients, carrying biallelic *TRIM32* variants (Table 1; Figure 1A).

As far as the literature review is concerned, the following terms were searched through PubMed in May 2023, filtering for human studies, abstract and full-text availability in English: “((trim32) AND (lgmdr8)) OR (limb-girdle muscular dystrophy recessive 8).” We included publications reporting patients of any age, and providing clinical, instrumental, and molecular characterization, with the latter clearly specifying the presence of *TRIM32* biallelic variants, either carried in homozygosis or compound heterozygosis. We applied exclusion criteria at both molecular and clinical levels: (1) the identification of monoallelic *TRIM32* variants led to the exclusion of the heterozygous patients; (2) among the patients carrying *TRIM32* variants biallelically, we excluded those cases clearly showing a non-LGMDR8 phenotype, such as the Bardet-Biedl syndrome type 11 (BBS11).

## 3 Results

### 3.1 Case report

A 63-year-old man came to our attention presenting with progressive muscle weakness and difficulty in climbing the stairs, since the age of 39. The patient was born to non-consanguineous parents, had a typical psychomotor development, and a negative family history of neuromuscular disorders. He stopped running in 12-month time from symptom onset, developing a waddling gait and inability to walk long distances by the age of 53. Serum CK levels were persistently increased over the years, ranging between 600 and 3,000 U/L (with 350 U/L representing the upper normal limit in men); his cardiopulmonary examination was always unremarkable.

**Figure 2:**
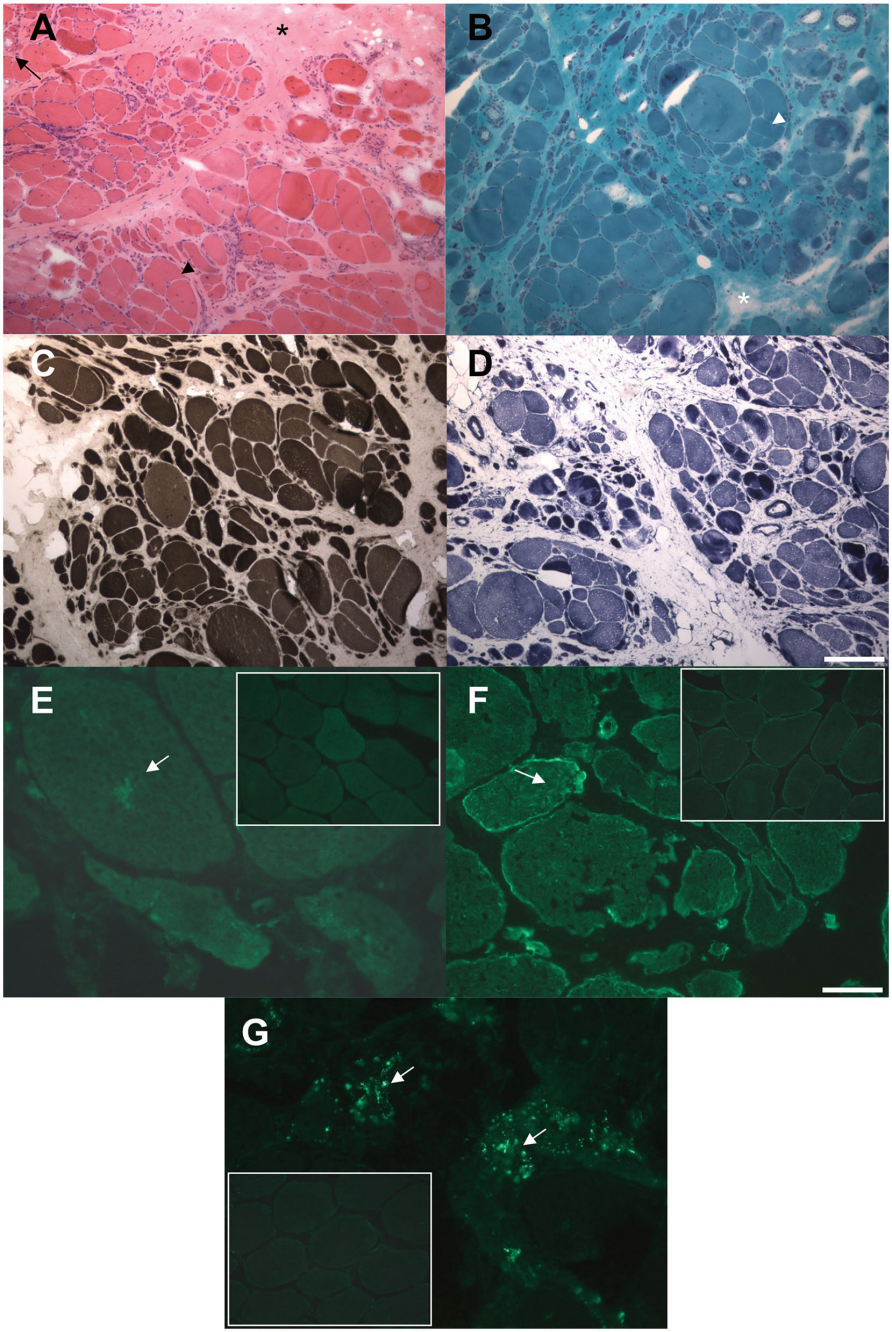
Histological and immunohistochemical findings in muscle biopsy. **(A, B)** Hematoxylin and eosin **(A)** and modified Gomori trichrome **(B)** stains showing markedly increased fiber size variability, central nuclei, severe fibro-adipose replacement (asterisks), splitting fibers (arrowheads), internal vacuoles (arrow). **(C)** ATPase pH 9.4 stain showing predominant type 2 (darker) fibers atrophy. **(D)** NADH-TR stain showing preserved oxidative enzymes activity in most of the observed fibers, with darker stain of the atrophic fibers. **(E, F)** Immunofluorescence assay showing increased intracellular accumulation of myotilin **(E)** and desmin **(F)** proteins compared to control muscles (small frames). **(G)** Immmunofluorescence assay showing increased cytoplasmic p62 signal compared to control muscle (small frame). Scale bars: 100 μm **(A–D)** 25 μm **(E–G)**.

A muscle biopsy of the vastus lateralis, collected at the age of 40, revealed dystrophic changes, such as severe fibroadipose replacement, predominance of type 2 fibers, increased fiber size variability, and presence of necrotic fibers with macrophagic invasion. Furthermore, there was evidence of numerous splitting fibers, subsarcolemmal and intracytoplasmic vacuoles containing finely granular material, without the presence of sarcotubular aggregates (Figures 2A–D). Immunohistochemistry showed desmin and myotilin accumulations within muscle fibers, as well as increased p62 detection compared to control muscles (Figures 2E–G). Electromyography (EMG) and electroneurography (ENG), performed at the early stage of the disease, showed a myopathic pattern of the quadriceps femoris, supporting the diagnosis of a pelvic girdle myopathy (clinical timeline is displayed in Figure 1B). The primary hypothesis of a putative involvement of the *DMD* gene, possibly responsible for Becker muscular dystrophy, was ruled out by Sanger sequencing and MLPA analysis, at the time of symptom onset.

His last physical examination revealed bilateral hypotrophy of the anterior compartment of the thigh and of the gastrocnemius, winged scapula, and selective weakness of pectoral, upper and lower limb muscles, causing waddling gait and bilateral foot drop. He was able to climb the stairs and stand up from a seated position only with the use of external aids. The patient reported to have recently experienced mild weakness of the upper limbs, and right thigh myalgias, with no cramps nor myoglobinuria. He did not complain of dysphagia or dysphonia. The most recent CK dosage was 2,400 U/L, in line with his previous values.

Following the neuromuscular visit, a muscle MRI scan was performed for the first time in 24 years of disease duration. This revealed diffuse morphological changes in several muscles of the upper and lower limbs (Supplementary Figure 1), with a severe fibroadipose degeneration involving the shoulder and pelvic girdles. In particular, the fatty infiltration pattern of the pelvic girdle and of the thigh muscles showed relative sparing of rectus femoris, gracilis, sartorius, and the short head of biceps femoris. The most affected compartments of the thigh were the medial and the posterior ones, with a diffuse bilateral hypotrophy, which was mostly symmetric. Dystrophic changes were also present at the level of the leg muscles, with relative sparing of the flexor hallucis longus, flexor digitorum longus and tibialis posterior muscles. As for the shoulder girdle, there was diffuse fatty degeneration, with relative sparing of the right supraspinatus, as well as of the trapezius, infraspinatus and pectoralis muscles, bilaterally. At the upper arm level, the most affected muscle was the triceps brachii, followed by the deltoid muscle. In contrast to what shown by the MMRI, clinical examination of the upper limbs was normal, with MRC score being 5 in all districts, except for pectoral muscles, where MRC score was 4 bilaterally.

**Figure 3:**
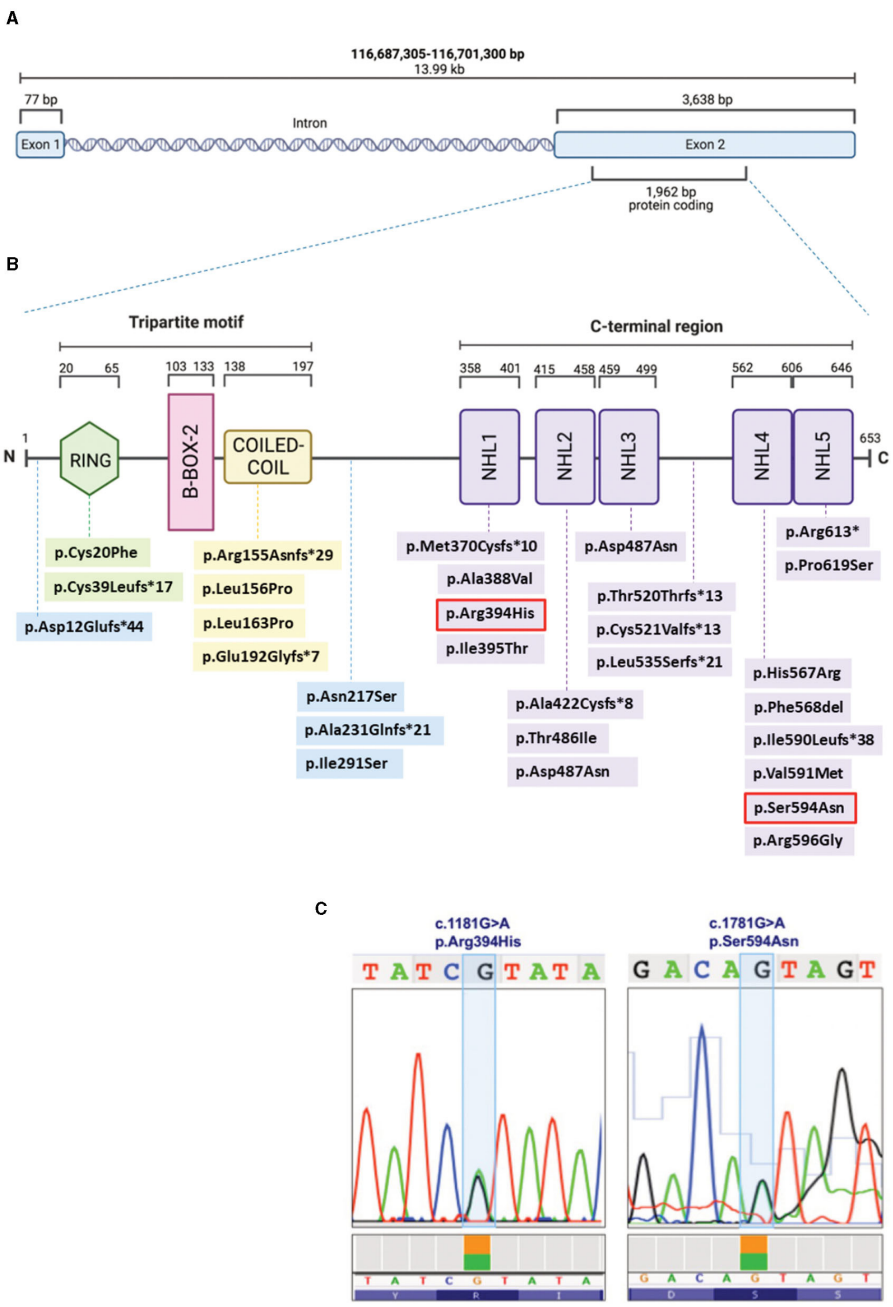
Schematic representation of the *TRIM32* gene **(A)** and TRIM32 protein **(B)**, displaying the structural domains and intermediate regions with the LGMDR8-associated biallelic variants (that are listed with the same color coding in Table 1). The two variants (c.1181G > A, p.Arg394His; c.1781G > A, p.Ser594Asn) carried by our patient are outlined in red **(B)** and also shown in the chromatograms **(C)**. Figures **(A, B)** were created with BioRender.com.

To make the definitive diagnosis of LGMDR8, a Clinical Exome Sequencing was performed at our center, revealing the presence of two heterozygous *TRIM32* variants (Figures 3B, C): c.1181G > A/p.(Arg394His), and c.1781G > A/p.(Ser594Asp). These were already described by Saccone et al. (11) and Panicucci et al. (25), and classified as class 4 (likely pathogenic) and class 3 (of uncertain significance) variants, respectively, according to the ACMG (American College of Medical Genetics and Genomics) criteria (Figure 3). Proband's parents or relatives were not available for segregation testing.

### 3.2 Literature review

We conducted a literature review of the clinical data related to 26 *TRIM32* variants, carried biallelically (in either compound heterozygosis or homozygosis) by 53 LGMDR8 patients (included our proband). Clinical and instrumental findings extracted from the 20 extant studies are reported in Table 1, and a visual summary of the LGMDR8 typical phenotype, based on the literature, is displayed in Figure 1A (7, 11, 13, 15–30).

The 91% (48/53) of the LGMDR8 patients described in the literature suffered from muscle weakness, usually self-reported as difficulty in walking/running and climbing the stairs. The upper limbs were involved in 71% of the patients (34/48), whereas proximal muscles (including those of the scapular girdle) were affected in 60% (29/48) of the cases. Other reported muscular symptoms were myalgias (11/53), exercise-intolerance (7/53), fatigue/fatigability (5/53), paresthesia (3/53), ankle contraction (3/53), muscle stiffness (1/53) and cramps (1/53). Physical examination also revealed lower limb (calves or quadriceps) hypertrophy in 13% (7/53) of the patients, and atrophy/hypotrophy (mainly affecting the lower limbs) in 32% (17/53) of them. Scapular winging was also reported in 13% (7/53) of the cases, and one patient presented with a scapuloperoneal phenotype (30). Finally, a small group of patients (5 out of 53), aged 4–33 years, did not complain of any muscle symptoms, and their neurological evaluation showed no signs of muscular impairment (7, 26). The clinical course showed progressiveness in 74% (39/53) of the cases. In this sub-group of progressive LGMDR8, 21% (8/39) of the patients needed unilateral (3/39) or bilateral (5/39) aids for walking, after 5–24 years of disease duration; while 23% (9/39) of them were wheelchair-bound at an age ranging from 30 to 70 years, after 8–40 years of disease progression. An EMG study was performed in 26% (14/53) of the LGMDR8 patients, who typically showed a myopathic pattern; neurogenic electromyographic changes were also reported in one-third of these cases.

Cardiac and respiratory involvement is not a frequent presentation in LGMDR8, but there might be cardiorespiratory alterations in some cases. Cardiological examination (electrocardiography and/or echocardiography) and spirometry testing were reported normal in 77% (30/39) of the patients with available cardiorespiratory data. The remaining 23% (9/39) showed some respiratory (10%, 4/39) and/or cardiac abnormalities (21%, 8/39), such as respiratory insufficiency and violations of myocardial conduction (Table 1).

MMRI studies of the lower limbs were described in 49% (26/53) of the cases [our patient; (13, 19, 21, 24–28)]. Their images showed fibroadipose degeneration, with preferential affection of the posterior thigh compartment and selective sparing of specific muscles. Upper limb MMRI findings have been rarely reported in the LGMDR8 literature [our case; (28)]. Wei at al. (28) described a minimal fatty infiltration without a distinct involvement pattern; conversely, our patient's MMRI showed diffuse fibroadipose replacement of the shoulder girdle, and involvement of the high arm, with selective sparing of specific muscles.

Histological findings were reported in 39 patients: 69% (27/39) of the biopsies showed nonspecific myopathic changes, whereas the remaining 31% (12/39) displayed a clearly dystrophic pattern, with two cases presenting both myopathic and neurogenic features. Immunohistochemical analysis of our patient's biopsy showed accumulations of desmin and myotilin, that are muscle-relevant targets of the TRIM32 ubiquitin ligase activity, alongside dysbindin, actin, α-actinin and tropomyosin (14). Similar findings were also observed in another LGMDR8 patient (25), who was homozygous for the c.1781G > A, p.(Ser594Asn) *TRIM32* variant, also carried by our proband. Furthermore, a certain degree of autophagy alteration has been observed in muscle samples from LGMDR8 patients: reduced levels of p62 and LC3II were described by Servián-Morilla et al. (26), whereas an increased p62 signal was detected in the muscle biopsy of our patient. Further investigations are therefore needed to elucidate TRIM32 pathways in autophagy regulation, and whether autophagy is up- or down-regulated in LGMDR8 (14).

## 4 Discussion

The TRIM family includes RING E3 ubiquitin ligases sharing a common evolutionary origin and a similar structure of the N-terminal RING domain as well as of the NHL repeats at the C-terminus.

In the TRIM32 653-amino-acid-long protein (Figure 3B), the N-terminal conserved tripartite motif consists of: (1) a RING structural domain (20–65 amino acids), which confers catalytic activity by interacting with the E2, and by promoting ubiquitin transfer; (2) a single type 2 B-box structural domain (103–133 amino acids), which possibly enhances the E3 RING domain activity (31) and modulates the rate of poly-ubiquitin chain synthesis (32); and (3) the Coiled-Coil structural domain (138–197 amino acids), which mediates dimerization and oligomerization (14). The C-terminus consists of five (or six) NHL repeats of about forty residues each (358–401, 415–458, 459–499, 562–605, 606–646), that are involved in protein binding and in mediating higher order self-association and homomultimerization (14).

Looking at the clinical features and genotypes of the 53 patients from our literature review, a clear genotype-phenotype correlation did not emerge. Interestingly, Guan and coauthors (20) recently analyzed 86 *TRIM32*-mutated patients from the literature, highlighting a correlation of the *TRIM32* variants exclusively impacting on the NHL domains, with a lower age at onset and higher CK levels, compared to subjects carrying variants outside the NHL regions. A gender effect was also observed, with a lower age of symptom onset, and higher CK levels, in males than in females (20). However, their analysis included both LGMDR8 and BBS11 patients, carrying bi- or monoallelic *TRIM32* variants (20), and this may explain the different results from our review, which instead specifically focused on LGMDR8-associated *TRIM32* biallelic variants.

To date, 50% (13/26) of the *TRIM32* variants (Table 1) considered to be causative for LGMDR8 (when present biallelically, either in homozygosis or in compound heterozygosis) are located in one of the five NHL repeats, like in the case of our patient. However, LGMDR8-causing *TRIM32* variants have been recently identified also in non-NHL domains (RING and Coiled-Coil), as well as in the intermediate regions outside the structural domains, like the pre-RING region (19), inter-CoiledCoil-NHL1 (26), and inter-NHL3-NHL4 (21). These findings dismantled the initial hypothesis of a selective involvement of the NHL domains in the LGMDR8 pathogenesis (14). In particular, the RING domain was found to be mutated (p.Cys20Phe, p.Cys39Leufs^*^17) in 4 LGMDR8 patients reported independently by Chakravorty et al. (17) and Servián-Morilla et al. (26). Additional 4 variants have been identified also in the Coiled-Coil domain (p.Arg155Asnfs^*^29; p.Leu156Pro; p.Leu163Pro; p.Glu192Glyfs^*^7) of other 7 LGMDR8 patients, described by Johnson et al. (21) and Mair et al. (22). The only TRIM32 structural domain which seems not to be involved in the LGMDR8 pathogenesis is the B-box-2. To date, the only noted B-box-2 variant is p.(Pro130Ser), which is, in fact, associated with BBS11 (33). BBS11 is a multisystemic disorder with no skeletal muscle involvement, characterized by obesity, polydactyly, retinal dystrophy and kidney abnormalities. Interestingly, despite LGMDR8 and BBS11 being two distinct disorders, some of the LGMDR8 patients (26) carrying the truncating variant, p.Cys39Leufs^*^17, in the RING domain, showed typical BBS11 systemic symptoms in addition to the LGMDR8 muscular presentation. A putative explanation for the presence of such different *TRIM32*-related phenotypes might be that RING and B-Box-2 domains have less muscular specificity and a more pleiotropic activity than the Coiled-Coil domain and the NHL repeats, that instead seem to mediate more muscle-specific functions.

Our patient carried a likely pathogenic variant in the NHL1 domain, and a variant of uncertain significance in the NHL5 domain. The NHL1 variant, p.(Arg394His), was previously reported in homozygosis in two LGMDR8 patients by Saccone et al. (11) and Johnson et al. (21); whereas the NHL5 variant, p.(Ser594Asp), was described in homozygosis in one patient by Panicucci et al. (25). Clinical, histological, and imaging features of our proband were very similar to the ones described in the patient carrying the NHL5 variant (25). As for the other two cases carrying the same NHL1 variant harbored by our patient, one had analogous muscular presentation and progressiveness of the disease (21), whereas the second patient, unlike our proband, presented with additional cardiorespiratory symptoms (11). Interestingly, two of these three cases were of Italian origins (11, 25), like our patient.

## 5 Conclusions

We presented a novel LGMDR8 patient of Italian origins, caused by two *TRIM32* missense variants for the first time described in compound heterozygosis, and previously reported in homozygosis in three independent patients (11, 21, 25). This case report provided new evidence in support of the pathogenicity of both p.(Arg394His) and p.(Ser594Asp) variants, that are not yet classified as pathogenic. We also defined the typical LGMDR8 phenotype associated with the so far identified 26 *TRIM32* variants, by performing a literature review of clinical and instrumental data related to 53 LGMDR8 patients. All these findings, taken together, might potentially be of help in improving both the diagnostic accuracy and the genetic counseling of this rare neuromuscular disease.

## Data availability statement

The raw data supporting the conclusions of this article will be made available by the authors, without undue reservation.

## Ethics statement

The studies involving humans were approved by Fondazione IRCCS Ca' Granda Ospedale Maggiore Policlinico. The studies were conducted in accordance with the local legislation and institutional requirements. The participants provided their written informed consent to participate in this study. Written informed consent was obtained from the individual(s) for the publication of any potentially identifiable images or data included in this article.

## Author contributions

MR: Data curation, Investigation, Writing—original draft. GR: Data curation, Investigation, Writing—original draft. FM: Investigation, Writing—review & editing. SA: Methodology, Visualization, Writing—original draft. CC: Investigation, Methodology, Writing—original draft. ES: Methodology, Writing—original draft. PC: Methodology, Writing—original draft. SZ: Methodology, Writing—original draft. DV: Methodology, Writing—original draft. SC: Writing—review & editing. GC: Writing—review & editing. DR: Conceptualization, Data curation, Investigation, Methodology, Supervision, Writing—original draft, Writing—review & editing.
