# Supplementary material for: Case report: A novel patient presenting TRIM32-related limb-girdle muscular dystrophy
Source: Front Neurol. 2024 Jan 18;14:1281953. doi: 10.3389/fneur.2023.1281953 (PMC10831852; doi:10.3389/fneur.2023.1281953)
Supplement: Supplementary file 1 [file Data_Sheet_1.pdf]

SUPPLEMENTARY MATERIAL

SUPPLEMENTARY FIGURE 1 (PAGE 1)
SUPPLEMENTARY METHODS (PAGE 2)

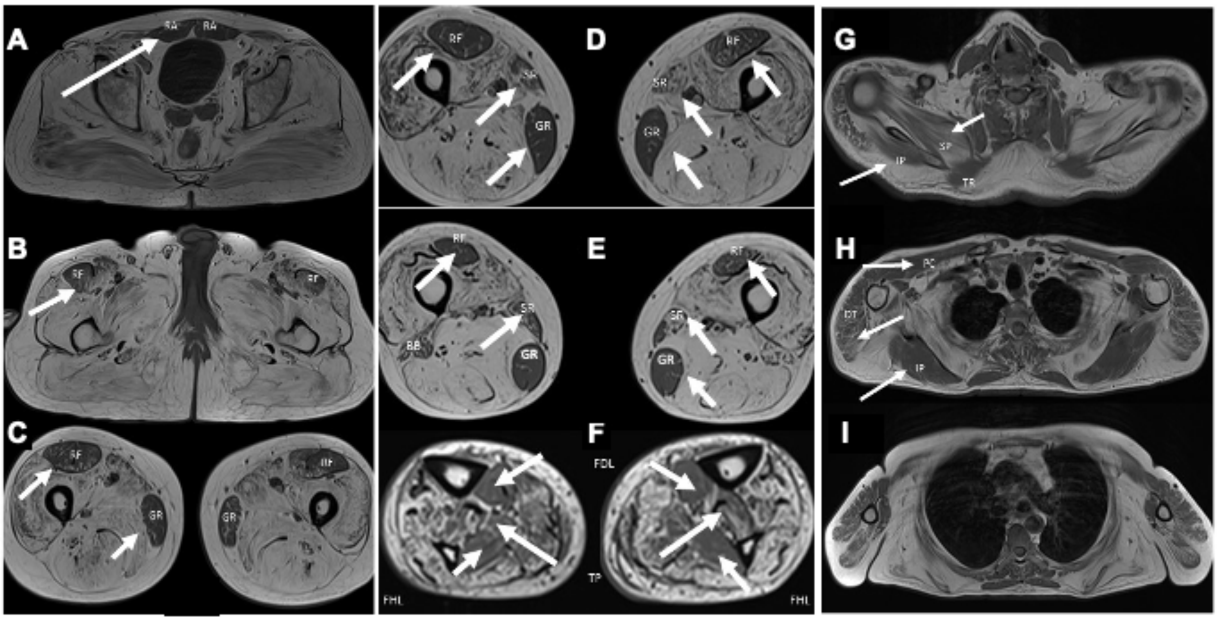

Supplementary Figure 1 Muscle MRI findings in our patient. T1-weithed axial MRI of lower limbs (A-F) and upper limbs (G-I) in our patient at 24 years of disease duration. White arrows indicate relatively spared muscles. FDL: flexor digitorum longus; FHL: flexor hallucis longus; TP: tibialis posterior; RA: rectus abdominis; RF: rectus femoris; GR: gracilis; SR: sartorious; BB: biceps femoris muscle short head; SP: supraspinatus; IP: infraspinatus; TR: trapezius; PC: pectoralis; DT: deltoid.

Supplementary Methods

The study was approved by the institutional review board of the Fondazione IRCCS Ca' Granda Ospedale Maggiore Policlinico, Milan (Italy). The patient provided written informed consent for all aspects of the study.

Histological and immunohistochemical studies

A biopsy of the vastus lateralis muscle was performed when the patient was 40. Tissue specimen was frozen in isopentane-cooled liquid nitrogen and processed according to standard techniques. For histological analysis, 8 μm-thick cryosections were picked and processed for routine staining with Haematoxylin and Eosin (H&E), Modified Gomori Trichrome (MGT), myosin ATPase (pH 9.4-4.6-4.3), cytochrome c oxidase (COX), succinate dehydrogenase (SDH), phosphatase acid, NADH, Oil Red O, Periodic Acid Schiff (PAS). Images fields were acquired at 40X using optical microscope Leica DM4000B equipped with DFC420C camera.

Muscle Magnetic Resonance Imaging studies

We performed Muscle Magnetic Resonance Imaging (MMRI) exam using a 1.5T Avanto Fit system, with TSE T1 (TR=653ms, TE=9ms, 5mm thickness) and STIR T2 (TR=3280 ms, TE=8.5ms, 5mm thickness) axial sequences on the pelvic girdle, thigh and leg, and TSE T1 (TR=722ms, TE=9 ms, 4 mm thickness) and STIR T2 (TR=3400ms, TE=8.5ms, 4mm thickness) axial sequences on shoulder girdle and upper arm bilaterally.

Molecular studies

Genomic DNA was extracted from peripheral blood, on a QiaSymphony Automated Nucleic Acid Extraction Platform (QIAGEN). Clinical Exome Sequencing was performed by using the Agilent Sureselect DNA target enrichment panel CCP17, according to manufacturer instructions. The obtained library underwent paired-end sequencing on a NextSeq500 Illumina platform. The variants included in the generated VCF files were annotated (according to the genome assembly of hg19) and classified according to an internal analysis pipeline (Ronchi et al., 2020), taking advantage of the Agilent Alissa platform.
The candidate variants in the *TRIM32* gene were validated by using PCR amplification, followed by Sanger sequencing (Thermo Fisher Big Dye Terminator v3.1) on an ABI Prism 3130 automated DNA analyzer.
